# MiR-96 induced non-small-cell lung cancer progression through competing endogenous RNA network and affecting EGFR signaling pathway

**DOI:** 10.22038/ijbms.2019.33654.8023

**Published:** 2019-08

**Authors:** Hao Ding, Mingqiang Chu, Jingjing Yue, Huaying Huang, Jian Wang, Li Zhu

**Affiliations:** 1 Division of Respiratory Disease, Affiliated People’s Hospital of Jiangsu University, Dianli Road No.8, Zhenjiang, 212002, China; 2 Division of Nephrology, Affiliated People’s Hospital of Jiangsu University, Dianli Road No.8, Zhenjiang, 212002, China

**Keywords:** DUSP1, EGFR, FOXO1, Metastasis, MiR-96, NSCLC

## Abstract

**Objective(s):**

Non-small cell lung cancer (NSCLC) has become a serious global health problem in the 21st century, and tumor proliferation and metastasis are the leading causes of death in patients with lung cancer. The present study aimed to verify the function of miR-96 and miR-96 in relation to competing with endogenous RNA regulatory network in NSCLC progression including proliferation and metastasis.

**Materials and Methods:**

Clinical data of miR-96 expression was collected from StarBase 2.0 developed by Sun Yat-sen University. We used wound-healing, transwell and MTT assays to measure migration, invasion and proliferation of NSCLC cell lines after different treatment. Quantitative real time PCR and western blot were used to test differential genes expression. In order to identify target between genes (FOXO1 and DUSP1) and miR-96, luciferase assay was used. Luciferase activities in FOXO1 and DUSP1 wild type plasmid groups were compared to mutant groups.

**Results:**

qRT-PCR and online database results indicated that miR-96 is highly associated with NSCLC when compared to normal patients. In addition, miR-96 indeed induced migration, invasion and proliferation of NSCLC cell line. In addition, FOXO1 and DUSP1 are targets of miR-96 and these three molecules form competing endogenous RNA network. miR-96 related competing endogenous RNA network affects cell metastasis via epidermal growth factor receptor (EGFR) signaling.

**Conclusion:**

miR-96 can be considered as one of tumor-inducer and form competing endogenous RNA network with FOXO1 and DUSP1, which affects downstream EGFR signaling.

## Introduction

Non-small cell lung cancer (NSCLC) including squamous cell carcinoma, and adenocarcinoma take up around 80-85% of all clinical cases of lung cancer (1). In addition, lung cancer has become a serious global health problem in the 21^st^ century as the incidence and mortality rate increased remarkably during the past decades. The 5-year survival rate still maintains under 20% even though the treatment efficacy has improved (2, 3). For NSCLC, tumor proliferation and metastasis are the leading causes of death in patients (4). Targeting molecular mechanisms of lung cancer is currently the main therapeutic approaches, especially for targeting aberrant epidermal growth factor receptor (EGFR) activity. Nevertheless, recent clinical treatments and researches explored resistance for EGFR targeting treatment (5). Therefore, identifying more detailed molecular mechanisms of NSCLC progressing is meaningful for solving drug resistance during clinical therapy.

MicroRNAs (miRNAs) are kind of non-coding RNAs with 18-24 nucleotides. MiRNAs regulate gene expression through binding to the miRNA response elements (MREs) on 3’ untranslational region (3’UTR) and affect mRNA stability or translation (6). Recent studies have found that miRNAs play important roles in tumorigenesis and progression (7, 8). Previous studies have shown that some of miRNAs function as tumor inducers in various cancer types (9-12). MiR-183, miR-155 and miR-9 induce NSCLC progression by targeting tumor suppressor genes (13-15). Among these miRNAs, miR-96 is now deliberated as it differentially expressed in cancer verified via Next Generation Sequencing. Zhang *et al.* (16) showed that miR-96 promoted proliferation of breast cancer cells, and meanwhile they detected that it has a highly expression level in breast cancer. In addition, miR-96 was found to promote cell proliferation and migration by targeting PTPN9 in breast cancer, and also it induces proliferation of lung cancer by targeting LMO7 (17). In NSCLC, the function and regulatory mechanism of miR-96 are not clear, hereby, we intend to uncover more about this miRNA function in NSCLC. In other studies, miR-96 combined with miR-145 and miR-9 impacts on peripheral blood mononuclear cells (18). According to competing endogenous RNA (ceRNA) hypothesis, miRNAs can target several different mRNAs together because of the same MREs on the sequences. Due to this ability, mRNA molecules may liberate other transcripts through competing miRNA binding (19). It is possible that miR-96 regulates NSCLC progression by forming ceRNA regulatory network together with other transcripts sharing common miR-96 MREs. Dual-specificity phosphatase 1 (DUSP1) has been found to inhibit migration and proliferation of gallbladder and lung cancer (20, 21). We connected this gene and transcription factor forkhead box O1 (FOXO1) with miR-96 in our study to give a detailed explanation on NSCLC progression. Ectopic expression of receptor tyrosine kinases (RTKs) has been proved to play a vital role in lung cancer, and recently, tyrosine kinase inhibitors targeting EGFR are hot spots of targeting therapy in variety of cancer types (22). However, patients with NSCLC who were initially responsive to EGFR inhibitors showed resistance (23). Thus, fully understanding the exact mechanisms of resistance need to be clarified urgently either. Our study aimed to verify miR-96 related ceRNA relationship with related genes in NSCLC and the affected EGFR signaling pathways by this network. 

## Materials and Methods

***Cell culture and clinical data***

Two NSCLC cell lines (A549 and PC-9) and one low invasive lung cancer cell line 95-C were purchased from American Type Culture Collection (ATCC) (Virginia, USA) and cultured with RPMI 1640 medium (Gibco, USA) according to instruction. HEK-293T cell line was cultured in L15 medium (Gibco, USA) with 10% fetal bovine serum (FBS, Gibco, USA). All cell lines were cultured in condition of 5% CO_2_ and 37 ^°^C. Clinical data of miR-96 expression level in different lung cancer patients were collected from online database StarBase 2.0 developed by Sun Yat-sen University (Website: http://starbase.sysu.edu.cn/panCancer.php). Searching criteria was set as clade: mammal; genome: human; assembly: hg19; microRNA: select miR-96; Number of supporting Experiments: default value; gene symbol: default value. Kaplan Meier plot was obtained from the database of kmplot (http://kmplot.com/analysis/)*.*

***Plasmid construction***

PmiR-Reporter plasmid was used to form FOXO1 and DUSP1 reporter plasmid in luciferase assay. PcDNA3.1 (+) was employed to build overexpression plasmid of FOXO1 and DUSP1. Primers of FOXO1 and DUSP1 for reporter plasmid and overexpression plasmid are listed below:

FOXO1: 5’ CAAACACTTCAGGACAATAA 3’

                                 5’ GTTCGCATAAACCACAATAC 3’

DUSP1: 5’ CCCCGAGAACAGACAAAGAG 3’

                                 5’ TGGCAACTAAAAAAAAACCC 3’

MiR-96 mimics and inhibitors were synthesized by GenePharma (Shanghai, China).

***Transfection***

Transfection was conducted according to lipofectamine 2000 protocol and previous study (24).

***Total RNA extraction and quantitative real time polymerase chain reaction***

Total RNA of each cell line was extracted using Trizol reagent and real time PCR was employed referring to the product instruction of real time PCR kit (Roche, Switzerland). Both of FOXO1 and DUSP1 were normalized to GAPDH. Quantitative real time polymerase chain reaction (qRT-PCR) was performed using an ABI7500 System (Applied Biosystems, CA, USA) and SYBR Green PCR Master Mix (TaKaRa, Dalian, China). The primers used are listed below:

FOXO1: 5’ GAGACCTCTGTAGTCCTGGG 3’

                                 5’ CTCAAAATGGTGAAGTGGAT 3’

DUSP1: 5’ GTTCCTCTGGGTTTCTAAGC 3’

                                 5’ GAGTCCTTTCTCTTCTGCCC 3’

GAPDH: 5’ GTCAACGGATTTGGTCTGTATT 3’

                                 5’ AGTCTTCTGGGTGGCAGTGAT 3’

***Wound-healing assay***

Briefly, sterile micropipette tip was used to scrap cell monolayer after transfection in 6-well plate. Cell debris was removed by washing with phosphate buffer solution (PBS). Following formula was used to calculate migration rate:

$\mathrm{migration rate}\left(\mathrm{\% of 0 hr}\right)=\frac{D_{0}-D_{T}}{D_{0}}\times 100\%$

Of which, D_0_ represents for distance at time beginning point and D_T_ represents for distance at 12 hr.

***Transwell assay***

Transwell assay was performed according to protocol provided by BD Biosciences. Briefly, 1×10^5^ cells were plated onto BD BioCoat Matrigel Invasion chambers (a pore size of 8 μm; BD Biosciences, USA) after suspending in serum free medium. Medium in lower chamber contained 10-15% FBS to form nutrient gradient. After 24 hr, the cells were stained with crystalline and then phase contrast microscopy was used to count cell numbers in at least five random fields.

***MTT assay***

A549 and PC-9 cells were plated into 96-well plate at the density of 5 × 10^3^ cells/well after transient transfected with miR-96 inhibitor and negative control. After incubation for 24, 48 and 72 hr, MTT assay was performed according to manufacturer’s instruction (Promega, USA).

***Luciferase assay***

To confirm microRNA targeting, HEK-293T cells and transient transfected with miR-96 mimics and DUSP1 or FOXO1 reporter plasmid were used. β-gal was co-transfected as an internal reference. Luciferase fluorescence was measured by microplate reader (Bio-Rad, USA) after treatment according to luciferase assay kit (Promega, USA). For wild or mutant type reporter plasmids of both genes, the detailed information of sequences was listed as following (5’ to 3’):

DUSP1-WT:AAAATACCAGTGTTGGGTTTTTTTTTAGTTGCCAACAGTTGTATGTTTGCTGATTATTT

DUSP1-MUT:AAAATACCAGTGTTGGGTTTTTTCGCGAGGTCCAACAGTTGTATGTTTGCTGATTATTT

FOXO1-WT:GCCATTGGAAATTTCATTACAATGAAGTGCCAAACTCACTACACCATATAATTGCAGA

FOXO1-MUT:GCCATTGGAAATTTCATTACAATGGCACATTCGCCTCACTACACCATATAATTGCAGA

***Western blot***

Total protein was extracted by using RIPA lysis buffer (Thermo Fisher, USA). For SDS-PAGE, 30 μg protein sample was loaded to separate. Separated proteins were transferred to PVDF membranes (Millipore, Germany) and then, membranes were incubated with anti-FOXO1 RabMAb (1:1000, Abcam, USA), anti-DUSP1 RabMAb (1:1000, Saier, China), anti-pEGFR RabMAb (1:1000, Abcam, USA), anti-MMP2 RabMAb (1:1000, Wanlei, China), and anti-MMP11 RabMAb (1:1000, Wanlei, China) over night at 4 ^°^C. Secondary antibody was used according to instruction and protein level was tested with ECL system (Thermo Fisher, USA).

***Drug application***

EGFR inhibitor AG1478 was purchased from APEXBIO, USA. Dimethyl sulfoxide (DMSO) was used as solvent and the final concentration was 20 μM for working solution according to product datasheet and the cells were incubated for 24 hr.

***Statistical analysis***

All results in this study were presented as mean±SD for three independent times. Difference between each group was compared by two side student’s t-test (*P-value < 0.05, **P-value < 0.01). 

## Results

***MiR-96 correlated with NSCLC***

**Figure 1:**
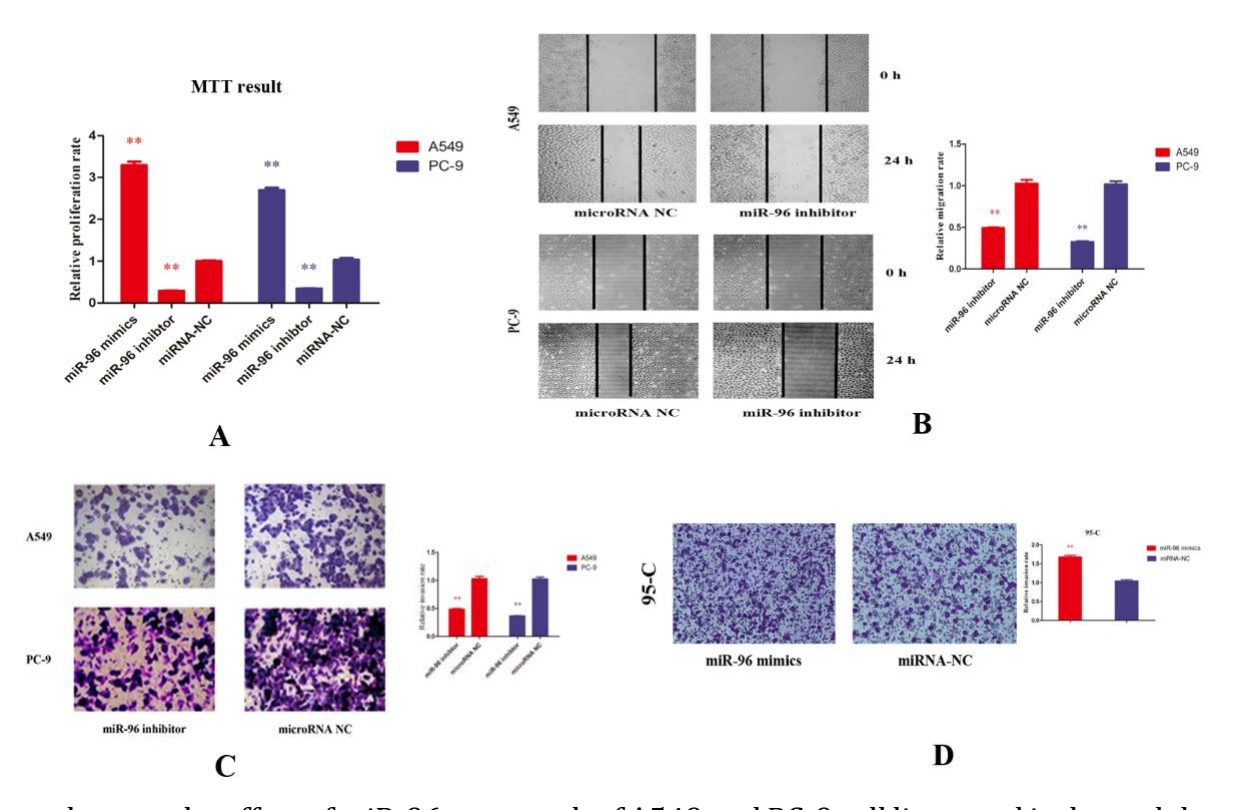
(A) miR-96 levels were measured by qRT-PCR in 3 lung cancer cell lines including A549, PC-9 and 95-C. miR-96 levels were normalized to U6 level in each cell line, and miR-96 in A549 is over 4 times (*P-value <* 0.01) compared to 95-C group, while miR-96 is over 3 times (*P-value <* 0.01) in PC-9. (B) and (C) We obtained clinical data of miR-96 expression in different lung cancer patients from the StarBase 2.0 powered by Sun Yat-sen University. In both lung squamous cell carcinoma (n=332) and lung adenocarcinoma (n=430) groups, miR-96 is highly expressed compared to normal group. *P-value<* 0.05, *P-value<* 0.01

qRT-PCR was employed to measure different miR-96 level in three different lung cancer cell lines including A549, PC-9 and 95-C. In Figure 1A, qRT-PCR showed that miR-96 correlated with invasive ability of lung cancer cells. Both A549 and PC-9 exhibited significantly higher miR-96 level compared to 95-C cell. In addition, this result is consistent with miR-96 expression profile in lung squamous cell carcinoma and lung adenocarcinoma provided by starBase v2.0, Sun Yat-sen University. In Figure 1B and 1C, high expression of miR-96 was observed in lung squamous cell carcinoma (n=332) and lung adenocarcinoma (n=430) compared to normal group (n=45, n=46 respectively). Accordingly, miR-96 can be observed as a biomarker of invasive NSCLC cells and is highly expressed in invasive lung cancer cells.

***Inhibition of miR-96 led to downregulated migration, invasion and proliferation in NSCLC cell lines***

**Figure 2:**
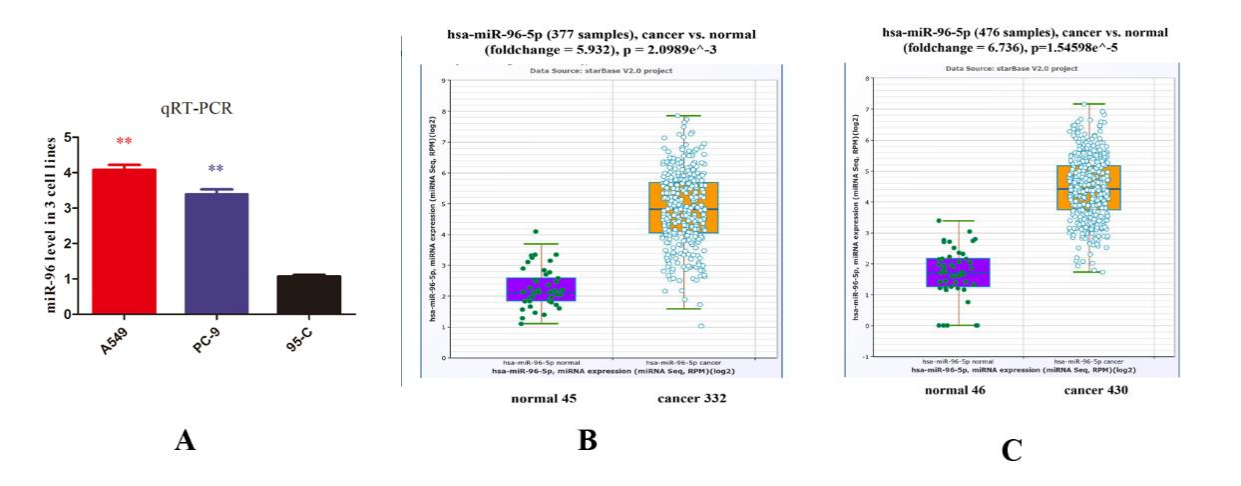
(A) MTT assay was used to test the effect of miR-96 on growth of A549 and PC-9 cell lines, and it showed that miR-96 inhibitor remarkably reduced cell growth rate of A549 and PC-9 cells, while miR-96 mimics induced it. (B) and (C) wound healing assay and transwell assay were employed and indicated that miR-96 inhibitor reduced migration and invasion ability of A549 and PC-9 cells after cultured for 24 hr. (D) For 95-C cell, miR-96 mimics also increased its invasion ability significantly measured by transwell assay. *P-value <* 0.01

A549 and PC-9 cells were treated with miR-96 inhibitor and corresponding negative control. MiR-96 inhibitor caused remarkably low cell growth rates in A549 and PC-9 cells compared to negative control group (Figure 2A). We further tested if miR-96 inhibitor had the same effect on migration and invasion. Wound healing assay (Figure 2B) showed that miR-96 inhibitor suppressed migration of A549 and PC-9 significantly, and Transwell assay (Figure 2C) provided proofs that miR-96 inhibitor suppressed invasion. Interestingly, miR-96 mimics increased invasion ability of 95-C cell (Figure 2D). Taken together, these results indicate that miR-96 exhibits a role of tumor induction and miR-96 inhibitor can reduce abilities of migration, invasion and proliferation of NSCLC cell lines.

***MiR-96 directly targets FOXO1 and DUSP1***

**Figure 3:**
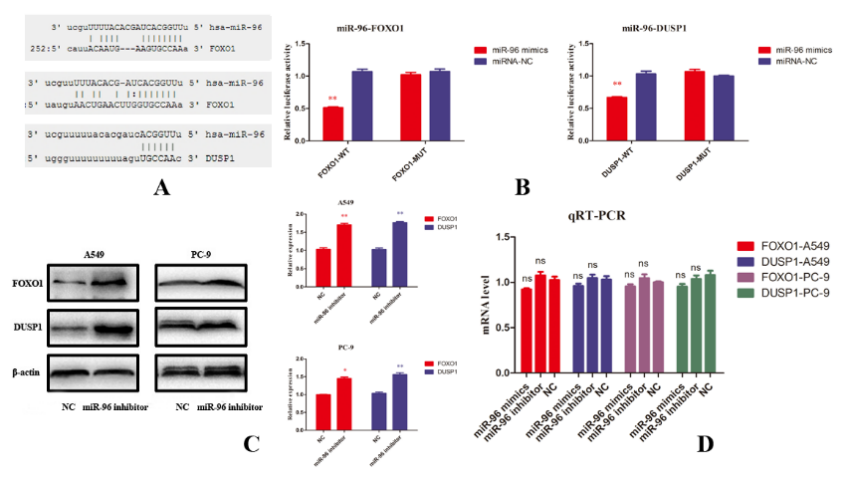
(A) Online prediction tool was used to find targeting relationship between miR-96 and related genes. It was predicted that miR-96 has binding sites on both forkhead box O1 (FOXO1) and dual-specificity phosphatase 1 (DUSP1) 3’UTR at different places. (B) Luciferase assay showed that miR-96 directly binds to both FOXO1 and DUSP1 measured by luciferase activity changings. (C) In A549 and PC-9 cells, after treatment with miR-96 inhibitor, FOXO1 and DUSP1 protein level increased significantly tested by western blot. (D) miR-96 cannot affect FOXO1 and DUSP1 mRNA level assessed by qRT-PCR. *P-value <* 0.05, *P-value <* 0.01

To clarify molecular mechanism of miR-96 inducing NSCLC progression, online prediction software TargetScan 2.0 was used to find potential miR-96 targets. Two targeted genes FOXO1 and DUSP1 with high scores were chosen for further study. As shown in Figure 3A, prediction results exhibited that there are two main targeted sites on FOXO1 3’UTR and one targeted sites on DUSP1 3’UTR. We constructed recombinant reporter plasmids containing targeted sequence to verify direct relations between miR-96 and these two genes. In Figure 3B, luciferase activity of FOXO1 and DUSP1 report plasmids were remarkably reduced by miR-96 mimics in HEK-293T cell when compared to miR-96 inhibitor and negative control. In addition, miR-96 inhibitor increased FOXO1 and DUSP1 protein levels in A549 and PC-9 cells (Figure 3C) but did not affect mRNA level of them (Figure 3D), which means that miR-96 regulates FOXO1 and DUSP1 through post-transcriptional modifications. Above data indicates that FOXO1 and DUSP1 are direct targets of miR-96 and implicates that miR-96 induces NSCLC progression by regulating these two genes.

***FOXO1 upregulated DUSP1 expression by sponging miR-96***

**Figure 4:**
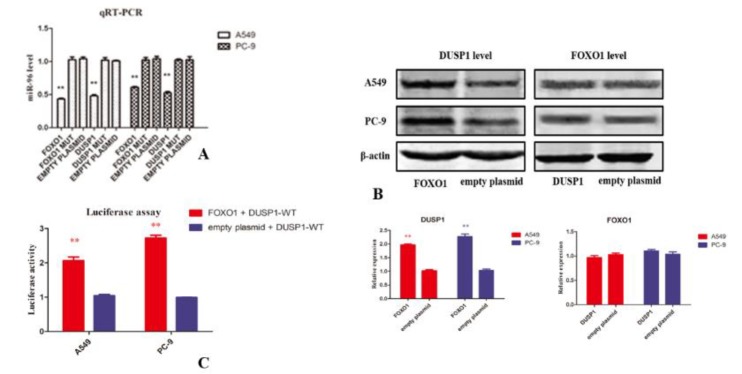
(A) The level of miR-96 decreased remarkably in groups transfected with forkhead box O1 (FOXO1) and dual-specificity phosphatase 1 (DUSP1) fragments, while their mutant sites cannot change it tested by qRT-PCR. (B) Western blotting showed that DUSP1 level increased when transfected with FOXO1 overexpression plasmid, while DUSP1 overexpression plasmid cannot change FOXO1 protein level. (C) FOXO1 increased luciferase activity of DUSP1 WT reporter plasmid. *P-value <* 0.01

Both FOXO1 and DUSP1 3’UTR have miR-96 targeted sites, thus we hypothesized that these three molecules can form a competitive endogenous RNA network that co-regulates NSCLC progression. Fragments containing FOXO1 and DUSP1 targeted sites or mutant sites were transfected into A549 and PC-9 cells. In Figure 4A, the level of miR-96 decreased remarkably in the groups transfected with FOXO1 and DUSP1 fragments, while their mutant sites could not change it, which suggests that both FOXO1 and DUSP1 function as sponges of miR-96. In addition, in Figure 4B, DUSP1 protein level was highly increased when treated with FOXO1 fragments in both cells, but DUSP1 could not affect FOXO1 protein level. DUSP1 report plasmid involving miR-96 sites were transfected into A549 cell together. Luciferase results in Figure 4C indicated that FOXO1 fragment led to a much higher luciferase activity of DUSP1 report plasmid when compared to empty vector. In summary, FOXO1 upregulated DUSP1 expression as a sponge of miR-96.

***EGFR signaling pathway can be affected by ceRNA network***

**Figure 5:**
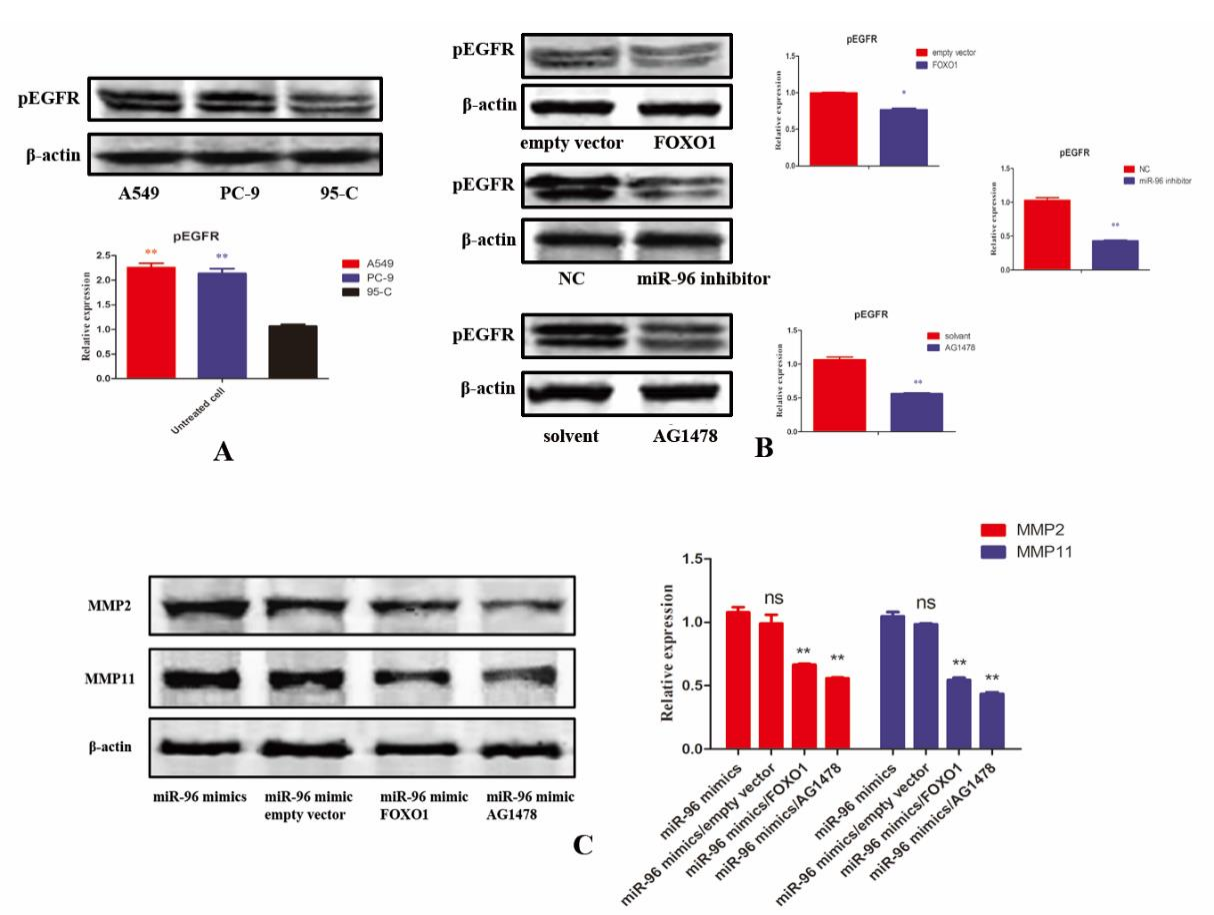
(A) Phosphorylated epidermal growth factor receptor (pEGFR) level in different lung cancer cell lines assessed by western blotting and its level in A549 and PC-9 cells is more than 2-fold higher than in 95-C. (B) Changing of pEGFR level when treated with forkhead box O1 (FOXO1) overexpression plasmid, miR-96 inhibitor and AG1478. All these three molecules can reduce pEGFR level. (C) Matrix metalloproteinases 2 (MMP2) and MMP11 level also decreased when treated with different agents or plasmid assessed by western blotting. *P-value <* 0.05, *P-value <* 0.01

As EGFR is highly associated with tumor migration and invasion, we tested effect of FOXO1-miR-96-DUSP1 axis on EGFR signaling. Much increased pEGFR level was observed in A549 and PC-9 cells when compared to 95-C cell in accordance with miR-96 level (Figure 5A). FOXO1 overexpression, miR-96 inhibitor and pEGFR inhibitor AG1478 abrogated pEGFR level significantly in comparison with empty vector, microRNA control and solvent in A549 cell (Figure 5B). To corroborate these data, two matrix metalloproteinases (MMPs) related to EGFR signaling were selected to test (25, 26). In Figure 5C, MMP2, and MMP11 were reduced by FOXO1, miR-96 inhibitor and AG1478, while miR-96 mimics can rescue their effect. Taking together, EGF signaling pathway is a downstream role, which can be regulated by FOXO1-miR-96-DUSP1 axis.

## Discussion

MicroRNAs are a group of molecules that function as regulators in every process of different cancers (19). Accordingly, miRNAs are considered as important therapeutic targets in cancer processing including apoptosis, proliferation, angiogenesis, cell migration and invasion. Cell signaling pathways are also involved in miRNA regulatory network as they can affect transcription factors, which are essential for protein members in signaling. Based on these properties, miRNAs provide researchers a variety of novel therapeutic possibilities.

In present study, we identified that miR-96 is an inducer of NSCLC development in 3 lung cancer cell lines. NSCLC cell lines have remarkably high level of miR-96 compared to 95-C cell, a type of pulmonary giant cell carcinoma cell with low metastatic ability. Clinical data from StarBase 2.0 also indicates that miR-96 is positively related to NSCLC. More importantly, inhibition of endogenous miR-96 in NSCLC cell lines reduced migration, invasion and proliferation of NSCLC cell lines. 

In human NSCLC cell lines, miR-96 functions through targeting two genes, DUSP1 and FOXO1. These three molecules are found to form a competing endogenous RNA relationship and DUSP1 locates on downstream of this network receiving regulation of FOXO1. Competing endogenous RNA hypothesis gives possibilities that numerous RNA sequences can be repressed by other different miRNAs because of common miRNA responding element. In this study, FOXO1 3’UTR plays a role of “sponge” absorbing endogenous miR-96, which leads to higher level of DUSP1 and reduces development of NSCLC. However, it still needs more detailed researches to uncover the preference binding for two sites of miR-96 on FOXO1. It probably depends on secondary structures of FOXO1 3’UTR.

In addition, EGFR signaling pathway is involved in this ceRNA regulation network. EGF peptide induces cell proliferation and metastasis with aid of EGFR (27). In this study, FOXO1 overexpression plasmid, miR-96 inhibitor and AG1478 had a similar function that abrogated pEGFR level significantly. EGFR-mediated expression of MMPs is upregulated in most cancers, which has crucial roles in invasion and metastasis (28, 29). EGFR and MMPs showed interconnectivity. Here, we tested MMP2 and MMP11 protein level, which further supported our points of FOXO1-miR-96-DUSP1 ceRNA relationship in EGFR signaling. However, we still need to notify the exact mechanism that how these three molecules bind each other and if there is any other unit takes part in the signaling pathway. As two miR-96 binding sites were predicted on the FOXO1 sequence, it is necessary to ensure if these two sites are equal for ceRNA relationship or secondary structure dependent. In several former studies, FOXO1 had an inhibitory effect on NSCLC migration. In addition, several miRNAs showed targeting relations with FOXO1, such as miR-183(13), miR-155(14) and miR-9(15). It indicates that FOXO1 is a truly potential target of miRNAs through which FOXO1 inhibits NSCLC migration. Although these studies clarified the functions of single gene in NSCLC, the complicated gene regulatory network is unclear yet. In our study, both FOXO1 and DUSP1 presented correlation with miR-96 that is considered as a tumor inducer in researches. It is the first time we connected three molecules that formed a network and uncovered how they co-regulate NSCLC metastasis and progression. 

## Conclusion

Taken together, our study revealed that miR-96 is a tumor inducer of NSCLC and forms ceRNA regulatory network with FOXO1 and DUSP1 genes to co-regulate NSCLC development depending on EGFR signaling pathway. However, we still need to know whether different target sites on FOXO1 have the same effect on sponging miR-96. As FOXO1 is one of important transcription factors, other genes in signaling pathways controlled by it should be considered into this co-regulatory network and provide a more comprehensive molecular mechanism of NSCLC progression.

## References

[1] Jemal A, Bray F, Center MM, Ferlay J, Ward E, Forman D (2011). Global cancer statistics. CA Cancer J Clin.

[2] Nishijima-Futami Y, Minami S, Futami S, Koba T, Higashiguchi M, Tamiya M (2017). Phase II study of S-1 plus bevacizumab combination therapy for patients previously treated for non-squamous non-small cell lung cancer. Cancer Chemothe Pharmacol.

[3] Zhu B, Yang J, Zhang P, Shen L, Li X, Li J (2017). Safety and effectiveness of localized lung resection combined with neoadjuvant chemotherapy in the treatment of stage I-II non-small cell lung cancer. Oncol Lett.

[4] Dong Y, Jin X, Sun Z, Zhao Y, Song X (2017). MiR-186 inhibited migration of NSCLC via targeting cdc42 and effecting EMT process. Mol Cell.

[5] Liu X, Wang P, Zhang C, Ma Z (2017). Epidermal growth factor receptor (EGFR): a rising star in the era of precision medicine of lung cancer. Oncotarget.

[6] Bak RO, Mikkelsen JG (2014). MiRNA sponges: soaking up miRNAs for regulation of gene expression. Wiley Interdiscip Rev RNA.

[7] Chakraborty C, Chin KY, Das S (2016). MiRNA-regulated cancer stem cells: understanding the property and the role of miRNA in carcinogenesis. Tumour Biol.

[8] Humphries B, Wang Z, Yang C (2016). The role of microRNAs in metal carcinogen-induced cell malignant transformation and tumorigenesis. Food Chem Toxicol.

[9] Dudics S, Venkatesha SH, Moudgil KD (2018). The micro-RNA expression profiles of autoimmune arthritis reveal novel biomarkers of the disease and therapeutic response. Int J Mol Sci.

[10] Fei X, Zhang J, Zhao Y, Sun M, Zhao H, Li S (2018). MiR-96 promotes invasion and metastasis by targeting GPC3 in non-small cell lung cancer cells. Oncol Lett.

[11] Wang Z, Yang B, Zhang M, Guo W, Wu Z, Wang Y (2018). lncRNA epigenetic landscape analysis identifies EPIC1 as an oncogenic incRNA that interacts with MYC and promotes cell-cycle progression in cancer. Cancer Cell.

[12] He C, Zhang Q, Gu R, Lou Y, Liu W (2018). MiR-96 regulates migration and invasion of bladder cancer through epithelial-mesenchymal transition in response to transforming growth factor-beta1. J Cell Biochem.

[13] Zhang L, Quan H, Wang S, Li X, Che X (2015). MiR-183 promotes growth of non-small cell lung cancer cells through FoxO1 inhibition. Tumour Biol.

[14] Hou L, Chen J, Zheng Y, Wu C (2016). Critical role of miR-155/FoxO1/ROS axis in the regulation of non-small cell lung carcinomas. Tumour Biol.

[15] Chen X, Zhu L, Ma Z, Sun G, Luo X, Li M (2015). Oncogenic miR-9 is a target of erlotinib in NSCLCs. Sci Rep.

[16] Zhang K, Wang YW, Wang YY, Song Y, Zhu J, Si PC (2017). Identification of microRNA biomarkers in the blood of breast cancer patients based on microRNA profiling. Gene.

[17] Hong Y, Liang H, Uzair Ur R, Wang Y, Zhang W, Zhou Y (2016). MiR-96 promotes cell proliferation, migration and invasion by targeting PTPN9 in breast cancer. Sci Rep.

[18] Budzinska M, Owczarz M, Pawlik-Pachucka E, Roszkowska-Gancarz M, Slusarczyk P, Puzianowska-Kuznicka M (2016). MiR-96, miR-145 and miR-9 expression increases, and IGF-1R and FOXO1 expression decreases in peripheral blood mononuclear cells of aging humans. BMC Geriatr.

[19] Thomson DW, Dinger ME (2016). Endogenous microRNA sponges: evidence and controversy. Nature Rev Genet.

[20] Shen J, Zhang Y, Yu H, Shen B, Liang Y, Jin R (2016). Role of DUSP1/MKP1 in tumorigenesis, tumor progression and therapy. Cancer Med.

[21] Shen J, Zhou S, Shi L, Liu X, Lin H, Yu H (2017). DUSP1 inhibits cell proliferation, metastasis and invasion and angiogenesis in gallbladder cancer. Oncotarget.

[22] Delaney C, Frank S, Huang RS (2015). Pharmacogenomics of EGFR-targeted therapies in non-small cell lung cancer: EGFR and beyond. Chin J Cancer.

[23] Huang L, Fu L (2015). Mechanisms of resistance to EGFR tyrosine kinase inhibitors. Acta Pharm Sin B.

[24] Yang J, Li T, Gao C, Lv X, Liu K, Song H (2014). FOXO1 3’UTR functions as a ceRNA in repressing the metastases of breast cancer cells via regulating miRNA activity. FEBS Lett.

[25] Zuo C, Li X, Huang J, Chen D, Ji K, Yang Y (2018). Osteoglycin attenuates cardiac fibrosis by suppressing cardiac myofibroblast proliferation and migration throughAntagonizing LPA3/MMP2/EGFR signaling. Cardiovasc Res..

[26] Du WW, Fang L, Li M, Yang X, Liang Y, Peng C (2013). MicroRNA miR-24 enhances tumor invasion and metastasis by targeting PTPN9 and PTPRF to promote EGF signaling. J Cell Sci.

[27] Tortora G, Bianco R, Daniele G, Ciardiello F, McCubrey JA, Ricciardi MR (2007). Overcoming resistance to molecularly targeted anticancer therapies: rational drug combinations based on EGFR and MAPK inhibition for solid tumours and haematologic malignancies. Drug Resist Updat.

[28] Mandel A, Larsson P, Sarwar M, Semenas J, Syed Khaja AS, Persson JL (2018). The interplay between AR, EGF receptor and MMP-9 signaling pathways in invasive prostate cancer. Mol Med.

[29] Majumder A, Ray S, Banerji A (2018). Epidermal growth factor receptor-mediated regulation of matrix metalloproteinase-2 and matrix metalloproteinase-9 in MCF-7 breast cancer cells. Mol Cell Biochem.

